# Ollivier Ricci curvature of directed hypergraphs

**DOI:** 10.1038/s41598-020-68619-6

**Published:** 2020-07-27

**Authors:** Marzieh Eidi, Jürgen Jost

**Affiliations:** 1 Max Planck Institute for Mathematics in the Sciences, Leipzig, Germany; 2 Santa Fe Institute, Santa Fe, New Mexico USA

**Keywords:** Applied mathematics, Mathematics and computing

## Abstract

Many empirical networks incorporate higher order relations between elements and therefore are naturally modelled as, possibly directed and/or weighted, hypergraphs, rather than merely as graphs. In order to develop a systematic tool for the statistical analysis of such hypergraph, we propose a general definition of Ricci curvature on directed hypergraphs and explore the consequences of that definition. The definition generalizes Ollivier’s definition for graphs. It involves a carefully designed optimal transport problem between sets of vertices. While the definition looks somewhat complex, in the end we shall be able to express our curvature in a very simple formula, $\kappa =\mu _0-\mu _2-2\mu _3$. This formula simply counts the fraction of vertices that have to be moved by distances 0, 2 or 3 in an optimal transport plan. We can then characterize various classes of hypergraphs by their curvature.

## Introduction

### Principles of network analysis

Network analysis constitutes one of the success stories in the study of complex systems^3,6,11^. For the mathematical analysis, a network is modelled as a (perhaps weighted and/or directed) graph. One can then look at certain graph theoretical properties of an empirical network, like its degree or motiv distribution, its assortativity or clustering coefficient, the spectrum of its Laplacian, and so on. One can also compare an empirical network with certain deterministic or random theoretical models. Successful as this analysis clearly is, we nevertheless see two important limitations. One is that many of the prominent concepts and quantities used in the analysis of empirical networks are node based, like the degree sequence. The structure of a network is encoded, however, not in its vertices or nodes, but rather in its edges, that is, the relations between the nodes. Therefore, here and in other works of our group, we wish to advocate and pursue an analysis whose fundamental ingredients are edge based quantities. Secondly, many real data sets are naturally modelled by structures that are somewhat more general than graphs, because they may contain relations involving more than two elements. For instance, chemical reactions typically involve more than two substances. This leads to hypergraphs, a subject that is currently gaining much momentum, see for instance^4^. In an undirected graph, an edge is given by an unordered pair of vertices, that is, by a two-element subset of the set of all vertices. For an undirected hypergraph, a hyperedge is given by any non-empty subset of the vertex set. In a directed graph, an edge is an ordered pair of vertices, that is, it connects two vertices, its tail and its head. Analogously, in a directed hypergraph, a hyperedge connects two non-empty sets of vertices, a tail set and a head set. Directed graphs thus are special directed hypergraphs, where each such set contains a single vertex. Like graphs, hypergraphs can also be weighted. Let us look at some examples. In a coauthorship network, the authors are the vertices, and a set of authors constitutes a hyperedge when they coauthor a paper. Thus, coauthorship networks are naturally modelled as undirected hypergraphs. Modelling them as a graph would connect two vertices when they are part of the set of authors of a paper. But already for three authors *A*, *B*, *C*, when they are pairwise connected in a graph, this cannot distinguish between the case where we have just three papers with two authors each, or whether there is a joint paper by all three of them, and perhaps in addition also some two or single author papers. Hypergraphs modelling empirical networks can also be directed. Taking the example of chemical reactions, they are typically not reversible, but rather transform a set of educts into a set of products. Thus, the task we set ourselves here is to forge tools that are powerful in the analysis of empirical networks modelled as hypergraphs. And as advocated above, such a tool should primarily evaluate properties of hyperedges rather than of vertices. Of course, once we have suitable quantities associated to hyperedges, quantities for vertices can be then also be derived, for instance by averaging over all hyperedges incident to or emanating from a vertex. So, how to go about this task? We take a conceptual approach and consider a hypergraph as a geometric object and then look for mathematical strategies for identifying invariants that can characterize geometric structures. And that leads us to one of the greatest successes in mathematics, Riemannian geometry. There, the fundamental invariants are curvatures. It might now seem that curvature is a concept only suitable for smooth structures, like smooth curves or surfaces, and therefore ill-suited for discrete structures like hypergraphs. But as it turns out, curvature concepts can be formulated more abstractly than taking second derivatives of some smooth objects. We shall now explain this in more detail, in order to motivate the approach taken in this paper.

### Ricci curvature: from Riemannian geometry to network analysis

In Riemannian geometry (see for instance^7^ as a reference), the curvature of a space quantifies its non-flatness. Among the various curvature notions that are of importance in Riemannian geometry, Ricci curvature quantifies this deviation by comparing the average distance between two sufficiently close points and the distance between two small balls around them. Bounds on curvatures can be used to connect the geometry of a Riemannian manifold with its topology, or to control stochastic processes on it. More precisely, a positive lower bound for the Ricci curvature yields the Bonnet–Myers theorem, which bounds the diameter of the space in terms of such a lower Ricci bound, the Lichnerowicz theorem for the spectral gap of the Laplacian, a control on mixing properties of Brownian motion and the Levy–Gromov theorem for isoperimetric inequalities and concentration of measures. In view of these strong implications, it is desirable to extend this to metric spaces that are more general than Riemannian manifolds. With this motivation, several generalized curvature notions have been proposed for non-smooth or discrete structures. In particular, Ollivier^12^ defined a notion of Ricci curvature on metric spaces equipped with a Markov chain, and extended some of the mentioned results for positively curved manifolds. His definition compares the Wasserstein distance between probability measures supported in the neighborhoods of two given points with the distance between these points. The Wasserstein distance between two probability measures is defined as the minimal cost needed for transporting one into the other. That is, an optimal transport problem has to be solved. On Riemannian manifolds, this recovers the original notion of Ricci curvature (up to some scaling factor), and at the same time, it naturally applies to discrete metric spaces like graphs. Recently, this curvature has been applied in network analysis, to determine spreading or local clustering in networks modelled by undirected or directed graphs, see for instance^14^. It is therefore desirable to have such a tool also for hypergraphs. In this paper, we shall develop a notion of an Ollivier-type Ricci curvature for, possibly directed and/or weighted, hypergraphs. There have been some prior proposals for extensions of Ollivier–Ricci curvature in such a direction (see for instance^1,2,15^), but our approach is more general and, as we argue, also more natural both in terms of its conceptual motivation and its range of applicability to empirical networks. From a geometric perspective, the fundamental principle that curvature characterizes types of spaces also applies here as we can distinguish and classify particular classes of directed hypergraphs in terms of their curvature. A definion of the Ollivier Ricci curvature of directed graphs was firstly proposed and investigated in^15^ where out-out directions for assigning measures are used. For that, however, one needs to assume strong connectivity of the underlying directed graphs in order to find transportation plans with finite cost, but this does not hold in many real directed networks. Therefore, in this paper, we work with in-out directions, which does not require such a strong assumption. The resulting theory is rather different from that of^15^. The first extension of the notion of Ollivier Ricci curvature to hypergraphs was proposed in^1^, using a multi-marginal optimal transport problem to define curvature. Because of that, the resulting curvature in the end is an analogue of Riemannian scalar rather than Ricci curvature. Also, it does not directly apply to directed hypergraphs. In this paper, we therefore propose a notion of directed hypergraph curvature that extends Ricci curvature rather than scalar curvature. Since in our setting, hyperedges are directed and each direction separates the vertices of the hyperedge into two classes, similar to directed graphs, we consider a double marginal optimal transport problem. We study some implications of our definition and then take a closer look at hypergraphs of constant Ricci curvature. In^9^, our notion of Ricci curvature for hypergraphs is systematically applied to empirical networks, leading to insight not readily available through other methods of network analysis.

### Ricci curvature

In order to proceed, we first need to explain the geometric meaning of Ricci curvature. Ricci curvature is a fundamental concept from Riemannian Geometry (see for instance^7^) that more recently has been extended to a discrete setting.

**Figure 1:**
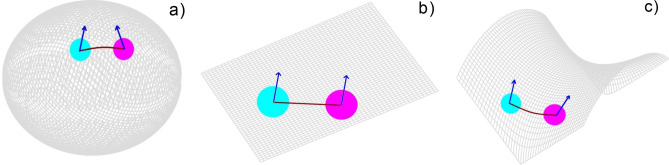
Manifolds with constant Ricci curvatures; (**a**) positive, (**b**) zero and (**c**) negative.

For a Riemannian manifold *M* of dimension *N*, Ricci curvature can be defined in several equivalent ways. What is relevant for the extension to the discrete setting is that it measures the local amount of non-flatness of the manifold by comparing the distance between two small balls with the distance of their centers when these centers are sufficiently close to each other. To be more precise, consider a unit tangent vector *w* at a point *x* in a Riemannian manifold M and let $\varepsilon ,\ \delta >0$ be smaller than the injectivity radius of *M*. Suppose $\exp _x(.) : T_x M\longrightarrow M$ denotes the exponential map and *y* is the endpoint of $\exp _x\delta w$ and hence at distance $\delta$ from *x*. Let $S_x$ be the sphere of radius $\varepsilon$ in the tangent space at *x* (and hence $\exp _xS_x$ is the sphere of radius $\varepsilon$ around *x* in the manifold). Then if $S_x$ is mapped to $S_y$ using parallel transport, the average distance between a point of $\exp _xS_x$ and its image in $\exp _yS_y$ is

$$\begin{aligned} \delta \left( 1-\frac{\varepsilon ^2}{2N}\mathrm {Ric}\ (w,w)+O(\varepsilon ^3+\varepsilon ^2\delta )\right) \end{aligned}$$

when $(\varepsilon ,\delta )\rightarrow 0$. If balls are used instead of spheres, the scaling factor is $\frac{\varepsilon ^2}{2(N+2)}$ instead of $\frac{\varepsilon ^2}{2N}$^13^. This follows from standard Jacobi field estimates. These estimates involve the sectional curvature, but summing over all directions orthogonal to the geodesic connecting *x* and *y* results in a Ricci curvature term. Here, one should think of $\varepsilon$ as being smaller than $\delta$, and $O(\varepsilon ^3)$ then simply indicates a higher term, whereas $O(\varepsilon ^2\delta )$ is needed when the Ricci curvature is not constant. In Riemannian geometry, one can then average the Ricci curvatures of the directions at a point. This then yields the scalar curvature, which thus is a quantity naturally associated to points. The scalar curvature is a much weaker geometric invariant than the Ricci curvature. Returning to the latter, if balls in average are closer than their centers (Fig. 1a), Ricci curvature in the direction of *xy* is positive. If the manifold is locally flat, Euclidian (Fig. 1b), then the two distances coincide. Most manifolds, however, are locally negatively curved (Fig. 1c)^10^.

This local characterization is the key property for defining Ricci curvature notions in more general settings than smooth manifolds. In 2007, Ollivier defined a notion of Ricci curvature, called Ollivier (coarse) Ricci curvature, on metric spaces equipped with a random walk *m*; Recall that if (*X*, *d*) is a Polish metric space equipped with its Borel $\sigma$-algebra, a random walk *m* on *X* is a family of probability measures $\{m_x|x\in X\}$ satisfying the following conditions^12^:

- The map $x\rightarrow m_x$ is measurable.
- Each $m_x$ has finite first moment, i.e., for some (hence any) $z \in X$ one has $\int d(z, y) dm_x(y) < \infty$.

Also in the following definition, we let *d*(*x*, *y*) be the distance from *x* to *y* obtained from the metric.

#### Definition 1.1^12^

Let (*X*, *d*) be a metric space with a random walk *m*, let $x,y\in X$ be two distinct points. The Ricci curvature of (*X*, *d*, *m*) in the direction (*x*, *y*) is

$$\begin{aligned} \kappa (x,y):=1-\frac{W_1(m_x,m_y)}{d(x,y)} \end{aligned}$$

where $W_1$ is the $1-$ Wasserstein distance between $m_x$ and $m_y$ on *X*:

$$\begin{aligned} W_1(m_x,m_y):=\inf _{{\mathcal {E}}\in \Pi (m_x,m_y)}\int _{(x,y)\in X\times X} d(x,y) \mathrm {d}{\mathcal {E}}(x,y) \end{aligned}$$

where $\Pi (m_x,m_y)$ is the set of measures on $X\times X$ whose first (second) marginal is $m_x$ ($m_y$) (thus, each ${\mathcal {E}}(x,y)$ is a coupling between random walks projecting to $m_x$ and $m_y$).

Here instead of taking metric balls around two close enough points we consider the Wasserstein distance (Transportation or Earthmover distance), between two probability measures $m_x$ and $m_y$ corresponding to two random walks which are starting at *x* and *y* respectively. When (*X*, *d*, *m*) is Riemannian manifold equipped with Riemannian volume measure, as it is shown in^12^, this notion coincides with the Riemannian Ricci curvature in the direction of *xy* (up to some scaling factor).

In network analysis, and particularly for networks that are modeled by undirected graphs, this notion has proved itself as a very useful tool to determine clustering and coherence in the network^8,14^, and since it is based on Markov chains, it is very well suited for capturing diffusion and stochastic processes in the network. One can see this as a motivation for the definition of^1^ where multi-marginal optimal transport was used to define a notion of curvature for collections of points in metric spaces. Since this is defined for points rather than for directions, from our perspective, this should be considered as a version of scalar curvature, rather than of Ricci curvature. However, the definition we shall present in the next section will be different.

## Results

### Transport plans and curvature of directed hypergraphs

As already mentioned, similar to directed graphs, in directed hypergraphs, every hyperedge *e* in *E* represents a directional relation between two non-empty subsets $$A_e$$ (tail), $$B_e$$ (head) of the vertex set *V*. In this paper we often write *A* instead of $$A_e$$, and *B* instead of $$B_e$$ when the hyperedge *e* is specified and fixed. Note that the sets $$A_e$$, $$B_e$$ and *V* can be equal, namely when the hypergraph has just one hyperedge *e* and its tail set and head set coincide. However if for a hyperedge *e*, $$ A_e$$ or $$B_e$$ is a proper subset of *V*, then since we consider non-empty subsets of *V*, the other one should also be proper. Similarly, for any vertex $$x\in V$$, $$d_{x}^{in}$$ is the number of incoming hyperedges to *x* (those hyperedges which include *x* in their head set), and $$d_{x}^{out}$$ is the number of outgoing hyperedges from *x* (those hyperedges which have *x* in their tail set). Also a directed path between the vertices in a directed hypergraph *G* is an alternating sequence of distinct vertices and directed hyperedges $$(v_1, e_1, \ldots , v_k, e_k, v_{k+1})$$ such that for each *i*, $$v_i $$ and $$v_{i+1}$$ are in the tail and head sets of $$ e_i$$ respectively. If $$ k\ge 1$$ and $$v_1$$ and $$v_{k+1}$$ are the same vertices, the path is called a directed *k*-cycle. From now on we consider directed hypergraphs to be weakly connected, meaning that the underling undirected hypergraph is connected.

#### Definition 2.1

Let $$H=(V,E) $$ be an unweighted directed hypergraph and $$e\in E$$ be an arbitrary directed hyperedge such that $$A=\{x_1,\ldots , x_n\}\xrightarrow {e}B=\{y_1,\ldots , y_m\}$$ $$(n,m \le |V|)$$. We define the Ollivier–Ricci curvature of this hyperedge as

$$\begin{aligned} \kappa (e):=1-W(\mu _{A^{in}},\mu _{B^{out}}) \end{aligned}$$

where the probability measures $\mu _{A^{in}}$ (called mass) and $\mu _{B^{out}}$ (called hole), are defined on *V* as follows:

$$  \begin{aligned} \mu _{A^{in}}= & {} \sum _{i=1}^n \mu _{x_i}^{in}\ \; {\text{where}} \ \; \forall 1\le i\le n\; {\text{and}}\; \forall z\in V(H)\\ \mu _{x_i}^{in}(z)= & {} {\left\{ \begin{array}{ll} 0 &{}\quad z=x_i \; \& \; d_{x_i}^{in}\ne 0\\ \frac{1}{n} &{}\quad z=x_i \; \& \; d_{x_i}^{in}= 0\\ \displaystyle \sum _{e';x_i\in B_{e'},\; z\in A_{e'}} \frac{1}{n \times d_{x_i}^{in} \times |A_{e'}|} &{}\quad z\ne x_i \; \& \; z\in A_{e'}\\ 0 &{}\quad  {\text{otherwise}} \end{array}\right. } \end{aligned}$$

and likewise

$$  \begin{aligned} \mu _{B^{out}}= & {} \sum _{j=1}^m\mu _{y_j}^{out} \ \; {\text{where}} \ \forall 1\le j\le m, z\in V(H):\\ \mu _{y_j}^{out}(z)= & {} {\left\{ \begin{array}{ll} 0 &{}\quad z=y_j \; \& \; d_{y_j}^{out}\ne 0\\ \frac{1}{m} &{}\quad z=y_j \; \& \; d_{y_j}^{out}= 0\\ \displaystyle \sum _{e';y_j\in A_{e'},\; z\in B_{e'}} \frac{1}{m \times d_{y_j}^{out} \times |B_{e'}|} &{}\quad z\ne y_j \; \& \; z\in B_{e'}\\ 0 &{}\quad {\text{otherwise}} \end{array}\right. } \end{aligned}$$

and $W(\mu _{A^{in}},\mu _{B^{out}})$ is the $1-$ Wasserstein distance between these two discrete measures defined as follows:

$$\begin{aligned} W(\mu _{A^{in}},\mu _{B^{out}})=\min _{{\mathcal {E}}\in \Pi (\mu _{A^{in}},\mu _{B^{out}})} \sum _{u\rightarrow A}\sum _{B\rightarrow v}d(u,v){\mathcal {E}}(u,v) \end{aligned}$$

where *d*(*u*, *v*) is the length of a shortest path from *u* to *v* and for a transport plan ${\mathcal {E}}$, ${{\mathcal {E}}(u,v)}$ represents the amount of mass moved from vertex *u* to vertex *v* and $\Pi (\mu _{A^{in}}, \mu _{B^{out}})$ is the set of transport plans, that is, probability measures on $V \times V$ that have $\mu _{A^{in}}$ and $\mu _{B^{out}}$ as their marginals. In other words, the minimum is taken over all couplings ${\mathcal {E}}$ between $\mu _{A^{in}}$ and $\mu _{B^{out}}$ which satisfy

$$\begin{aligned} \sum _{u\rightarrow A}{\mathcal {E}}(u,v)=\sum _{j=1}^m \mu _{y_j}^{out}(v)\text { and } \sum _{B\rightarrow v}{\mathcal {E}}(u,v)=\sum _{i=1}^n \mu _{x_i}^{in}(u) \end{aligned}$$

and by $A^{in}(u\rightarrow A)$ we mean the vertices $u \in V$ with $\mu _{A^{in}}(u) \ne 0$. Similarly $B^{out} (B\rightarrow v)$ refers to the vertices $v \in V$ with $\mu _{B^{out}}(v) \ne 0$.

#### Remark 2.1

Since we are working with directed objects, in this definition we had to provide for the situation where a vertex in the tail does not have incoming connections, or where a vertex in the head does not have any outgoing ones. In undirected networks, this is not an issue, because then the hyperedge in question itself provides both.

We can construct for each directed hypergraph a corresponding directed graph. That graph has the same set of vertices as the directed hypergraph and for each hyperedge, we draw an edge from each vertex in its tail to every vertex in its head. Thus, a directed hyperedge $A=\{x_1,\ldots , x_n\}\xrightarrow {e}B=\{y_1,\ldots , y_m\}$ corresponds to a set with *nm* elements of directed edges. Note, however, that there might be directed graphs that correspond to more than one directed hypergraph.

#### Proposition 2.1

*The curvature of a hyperedge* $e:A=\{x_1,\ldots ,x_n\}\rightarrow B=\{y_1,\ldots , y_m\}$*is bounded from below by the minimum of the Ricci curvatures of directed edges in its corresponding directed graph.*

#### Proof

Let ${\mathcal {E}}_{ij}$ be the optimal transport plan for the edge $e_{ij}:x_i\rightarrow y_j$, i.e.

$$\begin{aligned} W\left( \mu _{x_i}^{in},\mu _{y_j}^{out}\right) =\sum _{u,v\in V}d(u,v){\mathcal {E}}_{ij} (u,v) \end{aligned}$$

Then

$$\begin{aligned} {\mathcal {E}}:=\frac{1}{mn}\sum _{i=1}^n\sum _{j=1}^m{\mathcal {E}}_{ij} \end{aligned}$$

has the marginal distributions $\mu _{A^{in}}$ and $\mu _{B^{out}}$. Therefore:

$$\begin{aligned} W(\mu _{A^{in}},\mu _{B^{out}})\le \sum _{u,v\in V}d(u,v){\mathcal {E}} (u,v)=\frac{1}{mn}\sum _{i=1}^n\sum _{j=1}^m W\left( \mu _{x_i}^{in},\mu _{y_j}^{out}\right) \le \max _{\begin{array}{l} {1\le i\le n}\\ {1\le j\le m} \end{array}}W\left( \mu _{x_i}^{in},\mu _{y_j}^{out}\right) \end{aligned}$$

and therefore $$\kappa (e)\ge \min _{\begin{array}{l} {1\le i\le n}\\ {1\le j\le m} \end{array}}\kappa (e_{ij})$$. $$\square $$

However, the corresponding directed edges of a directed hyperedge do not fully represent the geometric structure of that hyperedge as shown in the following.

#### Remark 2.2

The maximum of the Ricci curvatures of directed edges corresponding to a directed hyperedge is not necessarily an upper bound for its Ricci curvature, as one can see from the example where the curvature of the hyperedge with red colour is one and the curvature of all its four corresponding directed edges is $-1/2$. 
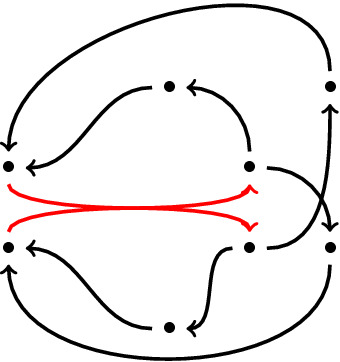


#### Lemma 2.1

*For a directed hyperedge* $e:A\rightarrow B$ *we have*

$$\begin{aligned} W(\mu _{A^{in}},\mu _{B^{out}})\ge \sup \left( \sum _{u\rightarrow A} f(u)\mu _{A^{in}}(u)-\sum _{B\rightarrow v}f(v)\mu _{B^{out}}(v)\right) \end{aligned}$$

*where the supremum is taken over all functions on* *V*(*H*) *with* $f(u)-f(v)\le d(u,v)$.

#### Proof

The proof is similar to the proof of proposition 2.10 in^15^ where another measure (out–out) for defining Ricci curvature of a directed edge is considered. Here we show that the same result holds for directed hypergraphs by assuming other directions (in–out) for defining measures. We have:

$$\begin{aligned} \sum _{u\rightarrow A}\sum _{B\rightarrow v} d(u,v){\mathcal {E}}(u,v)&\ge \sum _{u\rightarrow A}\sum _{B\rightarrow v} (f(u)-f(v)){\mathcal {E}}(u,v)\\ {}&=\sum _{u\rightarrow A} f(u)\sum _{B\rightarrow v} {\mathcal {E}}(u,v)-\sum _{B\rightarrow v}f(v)\sum _{u\rightarrow A}{\mathcal {E}}(u,v) \end{aligned}$$

thus:

$$\begin{aligned}&=\sum _{u\rightarrow A} f(u)\mu _{A^{in}}(u)-\sum _{B\rightarrow v}f(v)\mu _{B^{out}}(v) \end{aligned}$$

and since for all Lipschitz functions on hypergraphs this inequality holds and the left hand side is independent of *f*, we obtain

$$\begin{aligned} W(\mu _{A^{in}},\mu _{B^{out}})\ge \sup \left( \sum _{u\rightarrow A} f(u)\mu _{A^{in}}(u)-\sum _{B\rightarrow v}f(v)\mu _{B^{out}}(v)\right) . \end{aligned}$$

$$\square $$

#### Remark 2.3

In general, we do not get equality in this lemma; equality holds for undirected hypergraphs (see^15^), or more generally, if for every directed hyperedge *e* from $A_e$ to $B_e$, we have a directed hyperedge $e'$ in the reverse direction, from $B_e$ to $A_e$ (i.e, $A_{e'}=B_e$, $B_{e'}=A_e$). In that case, all distances *d*(*u*, *v*) become symmetric.

We now propose another formula for the curvature of a hyperedge which is more intuitive and in some cases much easier to work with.

For defining the Ricci curvature of a hyperedge *e*, we use incoming hyperedges to its tail set ($A_e$) and outgoing hyperedges from its head set ($B_e$). If *u* and *v* are in the support of $\mu _{A^{in}}$ and $\mu _{B^{out}}$ respectively, then $d(u,v)\le 3$. Recall that for any real valued function *f* on *V*, the support of *f* (denoted by *suppf*) is the set of all vertices in V where *f* is non-zero, i.e., ${supp} (f)=\{u\in V\,|\,f(u)\ne 0\}$.

If $\mu _i$ is the amount of mass that is moved with distance $i (i\le 3)$ in an optimal transport plan, then we have:

1 $$\begin{aligned} \sum _{i=0}^3 \mu _i=1, \quad \sum _{i=1}^3 i\mu _i= W . \end{aligned}$$

If $\kappa =0$ then $W=1$, and we thus have $\mu _0=\mu _2+2\mu _3$. More generally, we obtain

#### Theorem 2.1

*The curvature of a hyperedge is given by*

2 $$\begin{aligned} \kappa =\mu _0-\mu _2-2\mu _3. \end{aligned}$$

In fact, we could simply use (2) as a *definition* of Ricci curvature.The formula (2) for the curvature of a directed hyperedge, also works for curvature of edges in undirected graphs. As in the (undirected) graph case, $\mu _0$ represents the amount of mass which is not moved in an optimal plan, i.e., the amount of the stable mass in directed 3-cycles $(u\rightarrow x_i\rightarrow y_j\rightarrow u)$ (where *u* is simultaneously in the support of $\mu _{A^{in}}$ and $\mu _{B^{out}}$) or directed loops emerging from any of the $x_i$s. Although $\mu _1$ (the mass moved with distance one, possibly through directed quadrangles $(u\rightarrow x_i\rightarrow y_j\rightarrow v,$ while *u* is directly connected to *v*, $u\rightarrow v$) does not appear in the formula for the curvature, computing $\mu _1$ is an intermediate step for the computations of $\mu _2$ and $\mu _3$ where $\mu _2$ is the amount of mass that should be moved with distance 2 (possibly through directed pentagons including $x_i$ and $y_j$) and $\mu _3$ is the amount of the mass that is moved with distance 3 in an optimal plan.

#### Remark 2.4

While finding the general formula for $\mu _1$ (and $\mu _2$) may be difficult, any lower bound for $\mu _1$ (after simply knowing the exact amount of $\mu _0$) helps us to derive some upper bounds for *W* and therefore a lower bound for the curvature. Also the $\mu _i$ can differ between different optimal transport plans, but equalities (1) and (2) will always hold.

We also point out that while optimal transport plans always exist in our finite setting, they need not be unique.

**Figure 2:**
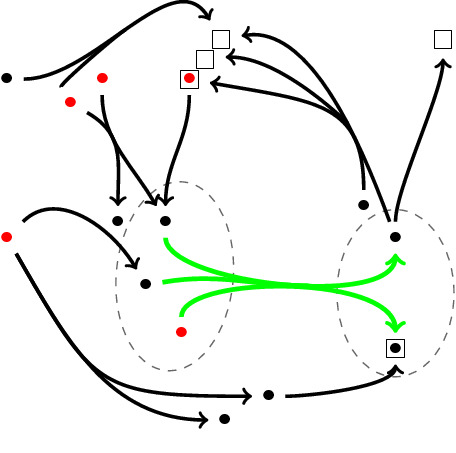
The green hyperedge is negatively curved.

As an example, in Fig. 2, for computing the curvature of the green hyperedge which connects three vertices in its left(*A*) to two vertices in its right(*B*), separated by dots, we assign masses (red bullets) and holes (empty squares) respectively to the incoming neighbours of *A* and outgoing neighbours of *B*. Note that since for a vertex *x* in *A* (the lowest vertex in *A*), $d_{x}^{in}=0$, we put all its assigned mass at *x* which is equal to 1/3. Similarly there is a vertex *y* in *B* such that $d_{y}^{out}=0$. Therefore we put its whole assigned measure at *y* itself and it is equal to 1/2. Also for the other vertices in *A* (respectively *B*), their assigned masses (holes) are divided between the vertices in the tails of incoming hyperedges to *A* (the vertices in the heads of outgoing hyperedges from *B*) based on Definition 2.1. It is straightforward to check that in (any) optimal transport plan, 1/12 of the mass need not be moved. This is the amount of the mass which coincides with one of the holes. Also 1/3 of the mass at *x* is moved with distance one to the hole at *y*. 1/6 is moved with distance two and the remained part is moved with distance three. Hence the curvature of the green hyperedge is $-11/12$.

### Bounds for the curvature

For obtaining an upper bound for the curvature of a hyperedge we need to control $\mu _0$ which corresponds to the stable mass at directed 3 cycles (triangles in the undirected graph case) and those vertices which are in the intersection of *A* and *B*.

In what follows, we shall see that increasing the number of vertices in this intersection will make the curvature more positive. Here, a directed hyperloop is a directed hyperedge $e:A=\{x_1,\ldots ,x_n\}\rightarrow B=\{y_1,\ldots , y_m\}$ for which $A\cap {B}$ is non-empty. Specifically where $A\cap {B}=A=B$, we shall have $\kappa (e)=1$. Directed *k*-cycles where $k \ge$ 4 do not affect the curvature of directed hyperedges since they can not make short-cuts for moving any of the masses to any of the holes.

#### Proposition 2.2

*For a directed hyperedge* $e:A=\{x_1,\ldots ,x_n\}\rightarrow B=\{y_1,\ldots , y_m\}$ *we have*

$$\begin{aligned} \sum _{u\in \mathrm {supp}\ \mu _{A^{in}}(u)\cup \mathrm {supp}\ \mu _{B^{out}} }\mu _{A^{in}}(u)\wedge \mu _{B^{out}}(u)\ge \kappa (e). \end{aligned}$$

*where* $$\alpha \wedge \beta :=min\lbrace \alpha , \beta \rbrace $$.

#### Proof

Jost and Liu have established this theorem in undirected graphs (thorem 7 in^8^). Here, we simply notice that the number of non-zero elements in this summation coincides with the number of vertices *u* belonging to a directed 3-cycle $(u\rightarrow x_i\rightarrow y_j\rightarrow u)$ or $A\cap {B}$. For other vertices *u* either of $\mu _{A^{in}}(u)$ or $\mu _{B^{out}}(u)$ is zero.

Also the equality holds when for a hyperedge e, all the masses coincide with all the holes with the same size. For instance in directed hyperloops when $A\cap {B}=A=B$ both sides of the above formula are equal to one. As another general example see Lemma 2.2. $\square$

As already mentioned in Remark 2.4 after computing $\mu _0$, any non-zero amount for $\mu _1$ would give us an upper bound for *W*. For that, at least one incoming neighbour of *A* should be at distance one from some outgoing neighbour of *B*. For example, when for the hyperedge e, $e:A=\{x_1,\ldots ,x_n\}\rightarrow B=\{y_1,\ldots ,y_m\}$, there is at least one hyperedge $e^\prime$ from any $y_j$ to any $x_i$ $(e^\prime :y_j\rightarrow x_i)$ or/ and when there is at least one $x_i$ with $d_{x_i}^{in}=0$ and at least one $y_j$ with $d_{y_j}^{out}=0$, the required condition is sattisfied and there is at least one mass which is in distance one from at least one hole and therefore we can present a transfer plan (similar to that in Theorem 3 in^8^) to obtain a positive lower bound for $\mu _1$. Specifically, in the same way that trees reach the smallest possible amount of curvature in undirected graphs, here hyperedges in directed hypertrees get the lowest possible number.

#### Definition 2.2

A directed loopless hypergraph is a hypertree if

1. There is at most one directed path between any two vertices and
2. It does not contain any directed cycle.

We note that although these two conditions are equivalent in undirected (hyper)graphs, they do not coincide in the directed case.

#### Theorem 2.2

*Let* $A=\{x_1,\ldots ,x_n\}$ *and* $B=\{y_1,\ldots ,y_m\}$ *be two subsets of vertices* *V* *of a hypertree* *H* *with respectively n and m elements and* $A\xrightarrow {e}B$ *be a hyperedge in this hypertree. If* *k* *elements in* *A* *have no incoming hyperedge, namely* $$\#\{ x_i \in A, d_{x_i}^{in}= 0\}=k\ $$ *and* $k^\prime$ *element in* *B* *have no outgoing hyperedge, i.e* $$\#\{ y_j \in B, d_{y_j}^{out}=0\}=k^\prime \ $$, *we have:* $\kappa (e)=-2+\frac{k}{n}+\frac{k^\prime }{m}$.

#### Proof

Note that since *e* is in a hypertree, according to the definition $\mu _0=0$. Hence in a hypertree, the curvature of any hyperedge is non-positive ($\kappa (e)\le 0$). We shall propose a transfer plan, which gives us an upper bound for *W*, and we will obtain a lower bound for *W* based on a single Lipschitz function (defined on the support of $\mu _{A^{in}}$ and $\mu _{B^{out}}$). We shall see that these two bounds coincide.

First we move $\left( \frac{k}{n}\wedge \frac{k^\prime }{m}\right)$ of the mass from *k* $x_i$’s to $k^\prime$ $y_j$’s with distance one. Then if $\frac{k}{n}\ge \frac{k^\prime }{m}$ we move $\frac{k}{n}-\frac{k^\prime }{m}$ of the mass from $x_i$’s with no incoming hyperedges to outgoing neighbours of the $y_j$’s with distance 2 and if $\frac{k^\prime }{m}>\frac{k}{n}$ we move $\frac{k^\prime }{m}-\frac{k}{n}$ of the mass at incoming neighbours of $x_i$’s to those $y_j$’s with no outgoing hyperedges with distance 2. Then we move the remaining part of the mass with distant 3. Therefore $W\le 3-\frac{k}{n}-\frac{k^\prime }{m}$.

On the other hand, for all *z* in *V*(*H*) we define

$$\begin{aligned} f(z)={\left\{ \begin{array}{ll} 3 &{}\quad \exists 1\le i\le n, \exists e:z\rightarrow x_i\\ 2 &{}\quad \exists 1\le i\le n, z=x_i\\ 1 &{}\quad \exists 1\le j\le m, z=y_j\\ 0 &{}\quad \text {otherwise} \end{array}\right. } \end{aligned}$$

It is straightforward to check that *f* is a Lipschitz function on $A\cup B\cup \mathrm {supp} \mu _{A^{in}}\cup \mathrm {supp} \mu _{B^{out}}$, hence according to the Theorem 3.5, we have

$$\begin{aligned} W(\mu _{A^{in}}, \mu _{B^{out}})&\ge \sup \sum _{z\rightarrow A}\mu _{A^{in}}(z)f(z)-\sum _{B\rightarrow z^\prime }\mu _{B^{out}}(z^\prime )f(z^\prime )\\ {}&\ge 3\left( 1- \frac{k}{n}\right) +2\left( \frac{k}{n}\right) -1\times \frac{k^\prime }{m}-0\times \left( 1-\frac{k^\prime }{m}\right) \\ {}&=3-\frac{k}{n}-\frac{k^\prime }{m}. \end{aligned}$$

and $$\displaystyle \kappa (e)=-2+\frac{k}{n}+\frac{k^\prime }{m}$$. $$\square $$

### Extension and reduction

- *Removing vertices from a hyperedge (Reduction)* In this part we want to investigate what happens to the curvature of an edge $$e:A=\{x_1,\ldots ,x_n\}\rightarrow B=\{y_1,\ldots , y_m\}$$ if we remove a number $$(l,l^\prime )$$ of vertices from *A* $$(l\le n)$$ and/or from *B* $$(l^\prime \le m)$$. Curvature depends on the connections between elements of $$\mathrm {supp}\ \mu _{A^{in}}$$ and $$\mathrm {supp}\ \mu _{B^{out}}$$ and removing different vertices from *A*(*and*/*orB*) might have different effects on the curvature. However since the amount of the masses (size of holes) which is assigned to any $$x_i$$(and $$y_j)$$ is already determined and is equal to respectively 1/*n* (and 1/*m*), we can give a bound for such changes.

#### Proposition 2.3

*Let* $e:A=\{x_1,\ldots ,x_n\}\rightarrow B=\{y_1,\ldots , y_m\}$. *By removing a vertex* $x_i$ *from* *A* *we get* $e^\prime : A-\{x_i\}\rightarrow B=\{y_1,\ldots , y_m\}$ *and we have*

$$\begin{aligned} |\kappa (e^\prime )-\kappa (e)|\le \frac{3}{n}. \end{aligned}$$

*Similarly, by removing* *l* *vertices from* *A* $(l<n)$ *we have*

$$\begin{aligned} |\kappa (e^\prime )-\kappa (e)|\le \frac{3l}{n} \end{aligned}$$

#### Proof

The two bounds for the curvature of $e^\prime$ arise from two extreme scenarios which might happen while removing vertex $x_i$ (or *l* vertices) from *A*.

1. If the whole mass which is assigned to $x_i$ is in directed loops or directed 3-cycles including $x_i$ and any of $y_j$ s, and after removing $x_i$, its corresponding mass has to be moved with distance 3 in an optimal plan, then
  $$\begin{aligned} {\left\{ \begin{array}{ll} \kappa (e)=\mu _0-\mu _2-2\mu _3\\ \kappa (e^\prime )=\left( \mu _0-\frac{1}{n}\right) -\mu _2-2\left( \mu _3+\frac{1}{n}\right) . \end{array}\right. } \end{aligned}$$
2. If the whole mass around $x_i$ was transported with distance 3 in an optimal plan and after removing this vertex, the corresponding mass is in the place of directed loops or directed 3-cycles including vertices of $A-\{x_i\}$ and *B*, then
  $$\begin{aligned} \kappa (e^\prime )=\left( \mu _0+\frac{1}{n}\right) -\mu _2-2\left( \mu _3-\frac{1}{n}\right) =\kappa (e)+\frac{3}{n}. \end{aligned}$$

Therefore we have

$$\begin{aligned} \kappa (e)+\frac{3}{n}\ge \kappa (e^\prime )\ge \kappa (e)-\frac{3}{n}. \end{aligned}$$

The same argument works for removing *l* vertices from *A* and the proof is complete. $\square$

Analogously, by removing $l^\prime$ vertices from *B* $(l^\prime <m)$ and using the same argument as before for the holes assigned to B, we have

$$\begin{aligned} |\kappa (e^\prime )-\kappa (e)|\le \frac{3l^\prime }{m}. \end{aligned}$$

Therefore:

#### Proposition 2.4

*By removinglvertices from the setAand* $l^\prime$*vertices fromB* $(e:A\rightarrow B)$*the following relation holds between the curvature of the resulting hyperedge* $(e^\prime )$*and the old one :*

$$\begin{aligned} |\kappa (e^\prime )-\kappa (e)|\le 3\left( \frac{l}{n}+ \frac{l^\prime }{m}\right) \wedge 3 \end{aligned}$$

- *Adding vertices to a hyperedge (Extension)* Here we want to obtain bounds for the curvature of a hyperedge obtained by adding some new vertices to the set *A* and/or to *B* and possibly adding new connections between them.

#### Proposition 2.5

*Let* $e:A=\{x_1,\ldots ,x_n\}\rightarrow B=\{y_1,\ldots , y_m\}$. *By adding* *l* *vertices to* *A* *and* $l^\prime$ *vertices to* *B* *we get a hyperedge* $e^\prime :A^\prime =\{x_1,\ldots ,x_{n+l}\}\rightarrow B^\prime =\{y_1,\ldots , y_{m+l^\prime }\}$ *with*

$$\begin{aligned} |\kappa (e^\prime )-\kappa (e)|\le 3\left( \frac{l}{l+n}+ \frac{l^\prime }{l^\prime +m}\right) \wedge 3 \end{aligned}$$

#### Proof

Here, since to each $x_i$ in $A^\prime$ we assign $\frac{1}{l+n}$ of the total mass $(=1)$ and $\frac{1}{l^\prime +m}$ of the total hole $(=1)$ is assigned to each $y_j$ in $B^\prime$, by considering the two extreme scenarios as before we have:

$$\begin{aligned} \mu _0(e)\rightarrow \mu _0(e^\prime )\pm \left( \frac{l}{l+n}+ \frac{l^\prime }{l^\prime +m}\right) \wedge 1 \end{aligned}$$

and therefore:

$$\begin{aligned} \mu _3(e)\rightarrow \mu _3(e^\prime )\mp \left( \frac{l}{l+n}+ \frac{l^\prime }{l^\prime +m}\right) \wedge 1 \end{aligned}$$

and according to the formula 2 the proof is complete. $\square$

#### Remark 2.5

Note that in Propositions 2.3, 2.4, 2.5, the equality holds when any of the mentioned extreme scenarios happen.

### Hypergraphs with constant Ricci curvature

We want to construct examples of directed hypergraphs in which the curvature of the hyperedges is constant $(\kappa =1,\kappa =0,\kappa =-2)$. In the case of $\kappa =0$ these (hyper)graphs are called Ricci flat. For brevity, we also call the others Ricci 1 and Ricci $-2$ directed hypergraphs.

- *Ricci 1 directed hypergraphs*

#### Theorem 2.3

*The vertices of a Ricci 1 directed loopless hypergraph which for every hyperedge* $e:\{x_1,\ldots ,x_n\}\rightarrow \{y_1,\ldots , y_m\}$ *does not have any hyperedge in the reverse direction* $(\not \exists e^\prime :y_j\rightarrow x_i)$, *can be divided into* 3 *subsets* *A*, *B*, *C* *such that* $A\rightarrow B\rightarrow C\rightarrow A$. *This means that some (not necessarily all) vertices in* *A* *are connected to vertices in* *B* *via a non-empty collection of directed hyperedges and similarly for the other connections as in the following diagram.*
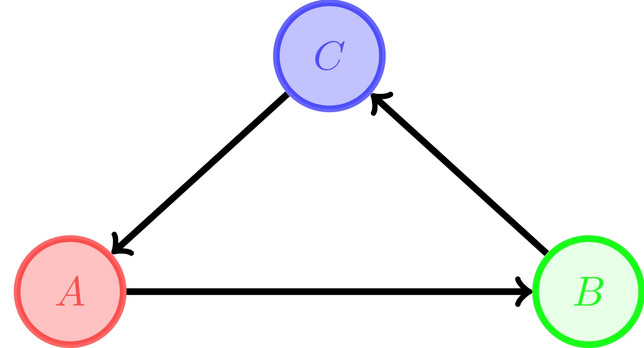


#### Proof

Consider a hyperedge $e_1:A_1\rightarrow B_1$. Since $\kappa (e_1)=1$

3 $$\begin{aligned} \mathrm {supp}\ \mu _{A_1^{in}}=\mathrm {supp}\ \mu _{B_1^{out}}=:C_1\quad \text {and}\quad \forall z\in C_1: \mu _{A_1^{in}}(z)=\mu _{B_1^{out}}(z) \end{aligned}$$

So the diagram related to $e_1$ looks like


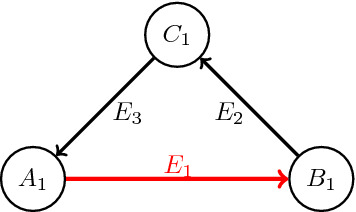
where $E_2$ and $E_3$ represent collections of directed hyperedges. Now, if there is no outgoing hyperedge from $C_1$ other than elements in $E_3$ and there is no incoming hyperedge to $C_1$ other than elements of $E_2$ and there is no outgoing hyperedge other than $e_1$ from $A_1$ and there is no incoming hyperedge to $B_1$ other than $e_1$, then $A_1,B_1$ and $C_1$ would be the desired partitioning set; Since the hypergraph is loopless, the intersection of any two of these sets is empty and because it is weakly connected the union of the vertices is the whole vertex set, V(H). If any of the above conditions does not hold, we can extend $A_1$ and/or $B_1$ and/or $C_1$ as follows:

For instance, let there be at least one hyperedge going out of $A_1$ other than $e_1$; we call it $e_{OA_1}:A_1\rightarrow B_{11}$ and we put $B_2=B_1\cup B_{11}$ where $B_1$ and $B_{11}$ are not necessarily disjoint. Since $\kappa (e_{OA_1})=1$, so $C_2:=\mathrm {supp}\ \mu _{B_{11}^{out}}=\mathrm {supp}\ \mu _{A_1^{in}}\supseteq C_1$. We next consider edges in $E_3$; If any of them has an endpoint outside $A_1$ and if the set of endpoints of $E_3$ is denoted by $A_2$, then $A_1\subseteq A_2$. By repeating this process we obtain an increasing sequence of $A_i$’s, $B_j$’s and $C_k$’s. We put $A=\cup A_i$, $B=\cup B_j$ and $C=\cup C_k$. Obviously, based on the process, elements in *A* are connected to *B*, *B* to *C* and *C* to *A* and these 3 sets are our desired partitioning. 
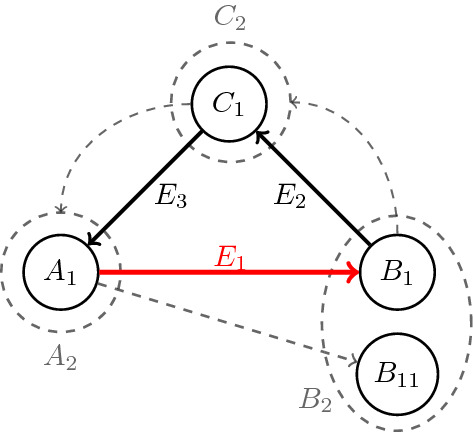
$$\square $$

#### Remark 2.6

The converse of this theorem is not necessarily true. For instance, the following hypergraph is not Ricci 1 although there is such a partitioning for the set of vertices of this hypergraph: 
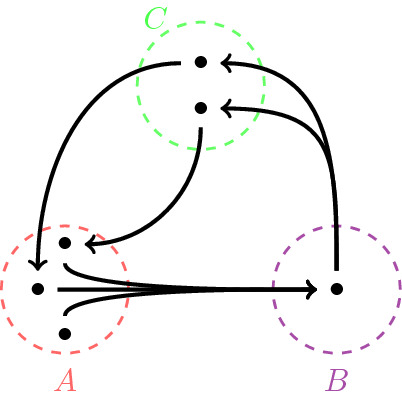


Instead we have the following:

#### Proposition 2.6

*If in the corresponding directed graph of a directed (loopless) hypergraph, the set of vertices can be partitioned into three different sets A, B, C such that* $A\rightarrow B\rightarrow C\rightarrow A$*and all of the elements inAare connected (via directed edges) to all the elements inBand similarly for the other arrows as above, then the original directed hypergraph is Ricci 1.*

Before proving this Proposition we state the next Lemma.

#### Lemma 2.2

*A directed (loopless) graph is Ricci 1 iff it’s set of vertices can be partitioned into 3 sets A, B, C such that* $A\rightarrow B\rightarrow C\rightarrow A$*and all the vertices inAare connected to all the vertices inBand similarly for the other arrows, as in the digram in previous theorem.*

#### Proof

$\Rightarrow$ The proof is similar to that of Theorem 2.3. Here, in addition all the vertices of *A* should be connected to all the vertices of *B* and so on. The reason is that here, for every edge $e:x\rightarrow y$, $d_{x}^{in}=d_{y}^{out}$ , and the condition that $\mathrm {supp}\ \mu _{x}^{in}=\mathrm {supp}\ \mu _{y}^{out}$ implies that the tails of incoming edges to *x* coincide with the heads of outgoing edges from *y*. Hence in the resulted partition every vertex in *A* is connected to every vertex in *B* and similarly for the connections between other sets the same situation holds.

$\Leftarrow$ For proving that every edge has curvature 1, for every edge $e:x\rightarrow y$, we should have $d_{x}^{in}=d_{y}^{out}$ and $\mathrm {supp}\ \mu _{x}^{in}=\mathrm {supp}\ \mu _{y}^{out}$ and for every *z* in this support $\mu _{x}^{in}(z)=\mu _{y}^{out}(z)$. Since in the partition all the vertices in *A* are connected to all the vertices of *B* and so on, for every edge the needed conditions obviously hold and the directed graph is Ricci 1. $\square$

#### Proof of Proposition 2.6

Since we have such a partition for the vertices of the corresponding directed graph of this hypergraph, according to the previous Lemma, the curvature of all the corresponding edges of each directed hyperedge is 1. Therefore their minimum also has curvature 1. On the other hand, according to Proposition 2.1

$$\begin{aligned} \kappa (\text {every hyperedge})\ge \min \kappa (\text {edges in the corresponding directed graph}) \end{aligned}$$

So for all hyperedges *e*, $\kappa (e)=1$ and the hypergraph is Ricci 1. $\square$

#### Remark 2.7

It might be possible that the directed hypergraph is Ricci 1, but as shown in the following example, its corresponding directed graph is not. 
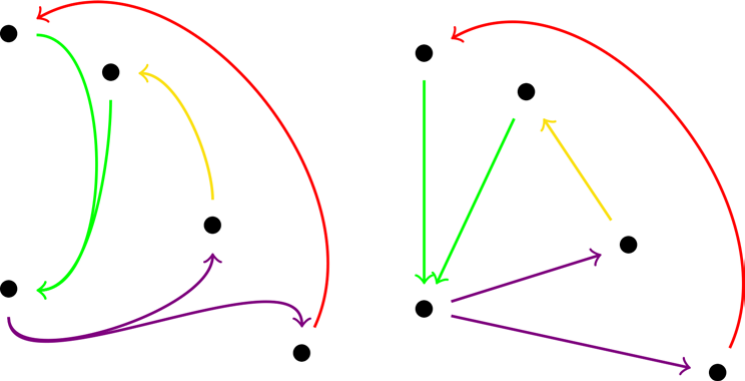

- *Ricci flat directed hypergraphs*

#### Theorem 2.4

*If the vertices of a directed hypergraph can be divided, as in the left diagram, into two sets* *A* *(source) and* *B* *(sink) such that all the vertices in* *A* *have outgoing hyperedges and no incoming hyperedges and all the vertices of* *B* *have incoming hyperedges and no outgoing hyperedges, then the hypergraph is Ricci flat . Also If the set of vertices can be divided into three sets*, *A*, *B*, *C* *such that all the vertices in these sets are connected to the vertices of the other sets as indicated in the right diagram, then the hypergraph is Ricci flat.*
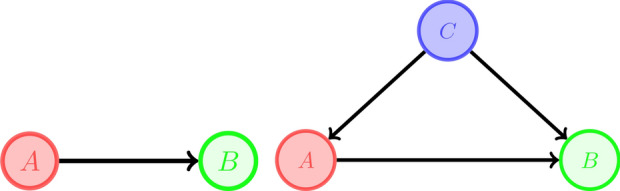


#### Proof

Based on the construction, for the two set partitioning, for every hyperedge, the masses are in the source set (*A*) which is at distance one from the holes which are in the sink set (*B*). So $\mu _1$ is equal to 1 and the hypergraph is Ricci flat . For the other case, since we are assuming that all the vertices of *A* are connected to all the vertices of *B* and similarly for other connections the same condition holds, for every hyperedge $e:A\rightarrow B$, the distance between any incoming neighbour of *A* to any outgoing neighbour of *B* is one. So $\mu _1$ is equal to 1 (and obviously by construction $\mu _0=0$). Therefore the hypergraph is Ricci flat . $\square$

#### Remark 2.8

The converse of the previous theorem is not necessarily true. However, If in a Ricci flat directed hypergraph for every directed hyperedge *e* there is no incoming hyperedge to its tail set and there is no outgoing hyperedge from its head set, then the set of vertices in this directed hypergraph can be partitioned into two sets *A* and *B* as above. That is because we can put all the tail sets of all of directed hyperedges in set *A* and all the head sets of all of directed hyperedges in set *B*.

In the above theorem, for 3-set partitioning, in contrast to the 2-set partitioning, the sets are partitioned into source, saddle and sink sets. The vertices in a saddle have both incoming and outgoing hyperedges. Also similar to the Ricci 1 case, connections inside any of these three sets might violate constant curvature along different hyperedges. As the last case, in the following part we introduce a class of directed hypergraphs with the most negative curvature.

- *Ricci negative* ($-2$) *directed hypergraphs*

#### Proposition 2.7

*If the set of vertices of a directed hypergraph can be divided into 4 sets*, *A* ,*B* , *C* *and* *D* *such that all the vertices in these sets are connected to the vertices of the other sets as indicated, then the hypergraph is Ricci* $-2$. *The presence of internal hyperedges (connections inside each of these sets) might violate constant curvature along different hyperedges.*
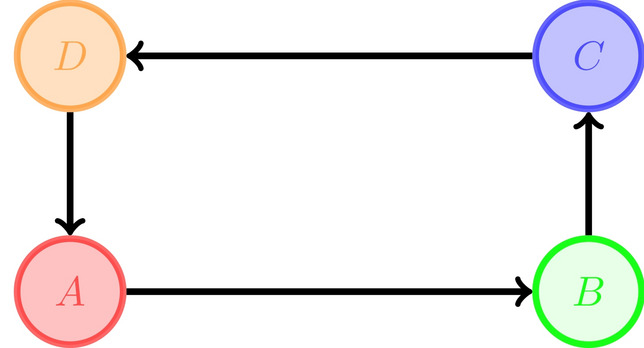


#### Proof

Here for every hyperedge $e:A\rightarrow B$, the distance between any incoming neighbour of *A* to any outgoing neighbour of *B* is 3. Therefore all $\mu _i =0$, except $\mu _3=1$, and every hyperedge has curvature $-2$. $\square$

### Weighted directed hypergraphs

We can extend our constructions to weighted directed hypergraphs where the vertices and hyperedges may both carry weights. The vertices may carry different weights depending on the hyperedges they are involved in (This can be represented by a vector of the dimension of the hyperedge set with non-negative components. Here, zero means the corresponding hyperedge does not involve that vertex). For a specified hyperedge whose curvature we want to measure, the weights of its vertices need to be fixed, of course. In this case we denote the vertex and hyperedge weights by $w_v$ and $w_e$, respectively

#### Definition 2.3

$$\begin{aligned} \kappa (e):=1-W(\mu _{A^{in}},\mu _{B^{out}}) \end{aligned}$$

where the probability measures $\mu _{A^{in}}$ (called mass) and $\mu _{B^{out}}$ (called hole), are defined on *V* as follows: Let $H=(V,E)$ be a weighed directed hypergraph and $e\in E$ an arbitrary directed hyperedge such that $A=\{x_1,\ldots , x_n\}\xrightarrow {e}B=\{y_1,\ldots , y_m\}$ $(n,m \le |V|)$. We define the Ricci curvature of this hyperedge as

$$\begin{aligned} \kappa (e):=1-W(\mu _{A^{in}},\mu _{B^{out}}) \end{aligned}$$

where the probability measures $\mu _{A^{in}}$ and $\mu _{B^{out}}$ are defined on *V* as follows:

$$  \begin{aligned} \mu _{A^{in}}= & {} \sum _{i=1}^n\mu _{x_i}^{in}\quad \forall 1\le i\le n \quad \text {and } \forall z\in V(H)\\ \mu _{x_i}^{in}(z)= & {} {\left\{ \begin{array}{ll} 0 &{}\quad z=x_i \quad \& \quad d_{x_i}^{in}\ne 0\\ \frac{w_{x_i}}{\sum _{i=1}^n w_{x_i}} &{}\quad z=x_i \quad \& \quad d_{x_i}^{in}=0\\ \sum _{e';z\in A_{e'},\; x_i\in B_{e'}}\frac{w_{x_i}}{\sum _{x_j\in A} w_{x_j}}\times \frac{w_{e^\prime }}{\sum _{e}w_{e:z\rightarrow A}}\times \frac{w_z}{\sum _{z \in A_{e'}}w_z}&{}\quad z\ne x_i\quad \& \quad z\in A_{e'}\\ 0 &{}\quad {\text{otherwise}} \end{array}\right. } \end{aligned}$$

Similarly, $$\mu _{B^{out}}=\sum _{j=1}^m \mu _{y_j}^{out}$$, $$ \forall 1\le j\le m, z\in V(H)$$

$$  \begin{aligned} \mu _{y_j}^{out}(z)={\left\{ \begin{array}{ll} 0&{}\quad z=y_j \quad \& \quad d_{y_j}^{out}\ne 0\\ \frac{w_{y_j}}{\sum _{y_i\in B} w_{y_i}}&{}\quad z=y_j \quad \& \quad d_{y_j}^{out}=0\\ \sum _{e';y_j\in A_{e'},\; z\in B_{e'}}\frac{w_{y_j}}{\sum _{y_i\in B} w_{y_i}}\times \frac{w_{e^\prime }}{\sum _{e}w_{e:B\rightarrow z}}\times \frac{w_z}{\sum _{z \in B_{e'}}w_z}&{}\quad z\ne y_j\quad \& \quad z\in B_{e'}\\ 0 &{}\quad {\text{otherwise}} \end{array}\right. } \end{aligned}$$

#### Remark 2.9

In weighted directed graphs, the Proposition 2.6 does not hold because, due to the weights we might have masses which coincide with holes of different sizes and therefore it violates Ricci 1 condition. For instance if we consider two directed 3-cycles which have one edge in common, by considering non-equal weights assigned to two other edges in the two cycles, the curvature of the common edge is not one although we have a three set partitioning in which all the required connections exist.

### Further differences between directed and undirected (hyper)graphs

**Figure 3:**
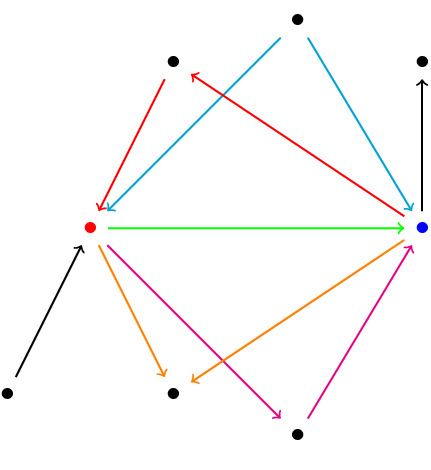
Directed triangles and their different impact on curvature.

- In directed (hyper)graphs, lower curvature bounds no longer control random walks.
- Since the Wasserstein distance no longer needs to satisfy a triangle inequality, we cannot define curvatures for vertex sets that are not connected by a hyperedge.
  These problems come essentially from the fact that we consider incoming edges at the tail *A* and outgoing edges at the head *B* of a hyperedge. In principle, we could of course also consider in-in or out-out relationships instead, but then, we might not always be able to move our masses, and so, curvatures might then become $-\infty$. This can only be avoided if we assume some strong connectedness condition in the directed case (see for instance^15^). Such a condition, however, is typically not satisfied in empirical data sets.
- The curvature of a directed (hyper)graph might be rather different from that of the underlying undirected (hyper)graph. For instance, in an undirected graph, every edge in a quadrangle has curvature zero. But a directed *k*-cycle where $k \ge 4$ is negatively curved.
- Directed triangles; For undirected graphs, it has been proven that the local clustering coefficient at each vertex can control the scalar curvature of any vertex which by definition is obtained by averaging over the Ricci curvature of all the edges connecting to that vertex (see Corollary 1 in^8^). In the undirected graph case, the local clustering coefficient is based on the number of triangles containing that vertex and its neighbours. In the directed case, after fixing the direction of every hyperedge, we encounter with four different types of triangles, bounded by only three edges, which share the property of having a directed edge that goes out from *A* and enters into the set *B*. Therefore in contrast to the undirected graph case, not all directed triangles but only some of them affect the curvature; in fact, for $\mu _0$ the presence of directed 3-cycles containing the vertices of *A* and *B* and of vertices *u* where $u\rightarrow x_i\rightarrow y_j\rightarrow u , u\in A^{in}(u\rightarrow A)$ and $u\in B^{out}(B\rightarrow u)$ increases the curvature of the corresponding hyperedge since they directly increase the amount of $\mu _0$. In contrast, those directed triangles which contain vertices of *A* and *B* and *u* in such a way that $x_i\rightarrow u, x_i\rightarrow y_j\rightarrow u , u\in A^{out}(A\rightarrow u)$ and $u\in B^{out}(B\rightarrow u)$ and those containing *A* and *B* and *u* such that $u\rightarrow x_i\rightarrow y_j, u\rightarrow y_j , u\in A^{in}(u\rightarrow A) , u\in B^{in}(u\rightarrow B)$ might affect on the amount of $\mu _2$ and thereby can make the curvature less negative. The last type of directed triangles are those which include vertices of *A* and *B*, outgoing vertices of *A* and incoming vertices to the set *B*, but they do not affect any of the $\mu _i$s and therefore the curvature. For instance in the directed graph depicted in Fig. 3, for computing the curvature of the green edge, the triangle consisting of the red and green edges affects $\mu _0$. Both triangles including orange-green and blue-green edges have an effect on $\mu _2$ and the curvature is not affected by the presence of the triangle of the pink-green edges.

## Conclusion

In contrast to ordinary graphs, hypergraphs can faithfully model higher order interactions between data points. Therefore, in empirical network science, nowadays graph representations gradually give way to hypergraphs. Under the label of Petri nets, directed hypergraphs have played an important role in computer science, but with an emphasis rather on processing schemes than on network properties. As systems get larger, however, network aspects are gaining importance^5^. Similarly, with the availability of large scale data sets, hypergraph methods are currently actively developed for the investigation of chemical reaction systems. Since we believe that instead of ad hoc schemes, one should rather develop a systematic mathematical theory, here we generalize concepts and constructions from Riemannian and metric geometry in order to provide such a theory. In particular, we have found that a version of the fundamental notion of Ricci curvature seems a natural tool to probe the local geometry of hypergraphs. Like the Ollivier Ricci curvature in graph theory, this curvature is defined in terms of optimal transport problem between probability distributions assigned to sets of vertices. Our notion can be used to differentiate between positively curved, flat and negatively curved hypergraphs. Consequently, in^9^, applying this tool to metabolic networks we have been able to identify patterns in these networks that can clearly distinguish them from random models that have been proposed as universal models in the literature. Thus, in this paper we demonstrate that a concept inspired from pure mathematics can be fashioned into a novel tool in applied mathematics for analysing systems of interacting elements.

## Data Availability

The datasets generated and/or analysed during the current study are available from the corresponding author on request.
